# Differential proteome response to H5N1 highly pathogenic avian influenza (HPAI) viruses infection in duck

**DOI:** 10.3389/fimmu.2022.965454

**Published:** 2022-08-19

**Authors:** Yu Ye, Huiying Fan, Qi Li, Zhen Zhang, Peisi Miao, Jun Zhu, Jie Liu, Jie Zhang, Ming Liao

**Affiliations:** ^1^ College of Veterinary Medicine, South China Agricultural University, Guangzhou, China; ^2^ College of Animal Technology and Science, Jiangxi Agricultural University, Nanchang, China; ^3^ National and Regional Joint Engineering Laboratory for Medicament of Zoonosis Prevention and Control, Guangzhou, China; ^4^ Key Laboratory of Animal Vaccine Development, Ministry of Agriculture, Guangzhou, China; ^5^ Key Laboratory of Zoonoses Control and Prevention of Guangdong, Guangzhou, China; ^6^ Key Laboratory of Control and Prevention of Guangdong Higher Education Institutes, Guangzhou, China; ^7^ Jiangsu Coinnovation Center for Prevention and Control of Important Animal Infectious Diseases and Zoonoses, Yangzhou, China; ^8^ South China Collaborative Innovation Center for Poultry Disease Control and Product Safety, Guangzhou, China

**Keywords:** H5N1 Highly Pathogenic Avian Influenza (HPAI) viruses, isobaric tags for relative and absolute quantitation (iTRAQ), host proteome response, mammalian target of rapamycin (mTOR) signaling, infection

## Abstract

Ducks and wild aquatic birds are the natural reservoirs of avian influenza viruses. However, the host proteome response that causes disease *in vivo* by the H5N1 HPAI virus is still unclear. This study presented a comprehensive analysis of the proteome response in Muscovy duck lung tissue during 3 days of infection with either a highly virulent DK383 or an avirulent DK212. An unbiased strategy- isobaric tags for relative and absolute quantitation (iTRAQ) in conjunction with high-performance liquid chromatography with tandem mass spectrometry (HPLC-MS/MS) was utilized to investigate the infection mechanism. Pathways derived from analysis of 292 significantly altered proteins may contribute to the high pathogenic nature and disease progression of H5N1 viruses. Global proteome profiles indicated improved correlation with the virus titers and gene expression patterns between the two strains of the H5N1 virus. DK383 replicated more efficiently and induced a stronger response specific to severe disease. While proteins involved in the immune response of neutrophils were increased markedly by DK383, DK212 evoked a distinct response characterized by an increase in proteins involved in the maturation of dendritic cells, adhesion of phagocytes, and immune response of macrophages. The differentially activated Akt/mTOR/p70S6K pathway might involve in the host response to H5N1 viruses. Therefore, systematically integrated with datasets from primary genomic and virus titer results, proteomic analyses may help reveal the potential pathogenesis.

## Introduction

The H5N1 HPAI viruses continuously circulate among poultry in parts of Asia and northeast Africa. Since October 2021, the H5N1 strain has swept across Africa, Asia, Europe, and North America, with around 3000 outbreaks reported (1). Occasionally, H5N1 viruses infect humans, causing disease with a mortality rate of nearly 60%. H5N1-infected wild birds (including ducks) do not usually display many signs of infection, which suggests that the duck has become the “Trojan horse” in spreading H5N1 (2). RIG-I, an influenza virus sensor absent in chickens, plays a role in the innate immunity of ducks against influenza. On the other hand, this provides a plausible explanation for ducks’ reduced susceptibility to influenza viruses compared with chickens (3). Many recent studies have focused on the pathogenicity of H5N1 HPAI viruses in animal models.

In 2002, there was a resurgence of the H5N1 strains that are highly pathogenic to ducks and cause death and severe neurological signs. This phenomenon may indicate that the biology of H5N1 influenza viruses in waterfowl is changing (4). Molecular mutation in some residues of the genome segment of H5N1 HPAI viruses has been demonstrated to be associated with lethality in ducks. For example, the amino acid changes of the polymerase genes PA (T515A) and PB1 (Y436H) are virulence factors (5). The substitution of two amino acids, S224P and N383D, in PA also contributes to the highly virulent phenotype (6). In addition, the HA and PA gene alteration induces high virus replication and an intense innate immune response in the brain (7). Although several virulence factors have been identified, host factors are also responsible for the pathogenesis of infections. Elevated inflammatory cytokine and chemokine production break the homeostasis of the respiratory and immune system during influenza virus infection, whereas macrophages that migrate to influenza-infected lungs play a pathogenic role in pulmonary inflammation (8). However, the studies on the host response of ducks to H5N1 influenza viruses have been far from optimal.

Previous work analyzing the host response of ducks to H5N1 influenza virus infection has primarily utilized *in vitro* systems (9). Moreover, *in vivo* studies of molecular differences in host response are confined only to RNA expression profiling analogous to *in vitro* studies (7, 10, 11). Unfortunately, little work has been done in proteome profiling to investigate the host-virus interaction within the duck model.

Our study presents a comprehensive analysis of the host proteome response to influenza virus infection based on an unbiased strategy-iTRAQ in conjunction with HPLC-MS/MS. Muscovy ducks were infected with either a highly virulent (A/Duck/Guangdong/383/2008, DK383) or an avirulent (A/Duck/Guangdong/212/2004, DK212) H5N1 HPAI virus. Lung tissue was then collected to evaluate viral titers 1, 2, and 3 days post-infection (dpi). While DK383 caused death between 3 and 4 dpi, DK212 in ducks appears asymptomatic. Based on the information presented above, the lung tissue at 3 dpi was chosen to perform proteomic analysis. 2,459 proteins were quantified, including 292 significantly altered proteins during productive infection. Functional analysis integrated with previous observation in our laboratory (10) provides a complete view of the host proteome response and in-depth characterization of viral pathogenesis.

## Materials and methods

### Virus, cells, and animals

The H5N1 HPAI viruses DK383 and DK212 were isolated in the Guangdong province of China (12). Viral titers of influenza stock were measured by plaque assay to prepare for duck embryonic fibroblasts (DEF) infection (13).

Fibroblasts were extracted from 12-day-old Muscovy duck embryos. Cells were plated into cell culture flasks and maintained overnight in DMEM with 10% fetal bovine serum, 100 U/ml of penicillin, and 100 μg/ml of streptomycin at 39°C, 5% CO_2_.

MDCK (Madin-Darby canine kidney) cells were grown in DMEM supplemented with 10% fetal bovine serum at 37°C and 5% CO_2_.

One-day-old healthy Muscovy ducks were purchased from a commercial hatchery in Guangzhou. Congenitally avian influenza-negative ducks were chosen by agar gel precipitation tests and hemagglutinin inhibition (HI) assays to be experimental animals. All experiments were carried out in Animal Biosafety Level 3 (ABSL-3) facilities.

### Infection of ducks and cells

At 4 weeks old, 42 ducks were randomly assigned into 3 groups (DK383, DK212, and mock control). In each group, ducks were inoculated by the intranasal route with 10^6^ EID_50_ in 0.2 ml of either DK383 or DK212 and 0.2 ml phosphate buffered saline (PBS) as mock controls. After the challenge, ducklings were observed for 14 days for clinical signs of infection. Three ducks from each group were sacrificed at 1, 2, and 3 dpi. Their lung tissues were collected and inoculated into SPF eggs, and virus titers were determined as previously described (10). All animal experiments were conducted following the guidance of CDC’s Institutional Animal Care and Use Committee and in an Association for Assessment and Accreditation of Laboratory Animal Care International-accredited facility. The protocol of animal experiments in this study had been approved by the ABSL-3 Committee of South China Agricultural University.

DEF cells were infected with avian influenza viruses at a multiplicity of infection (MOI) of 0.01. Cells inoculated with serum-free DMEM were used as mock-infected controls. After incubation for 1 h, the cells were washed once with PBS, cultured with maintenance media at 39°C at 5% CO_2_, and harvested at 12 hpi.

### Protein preparation and iTRAQ labeling

Lung tissue was collected and washed with cold PBS. Proteins were extracted by a RIPA reagent containing a complete protease inhibitor. After vibrating several times, the lysate was sonicated for 8 cycles of 1 s on and 1 s off on ice with a JY96-II (Ningbo Scientz Biotechnology), then centrifuged at 12,000 g at 4°C for 10 min. Protein concentration was measured through BCA Protein Assay Kit (Thermo Scientific). Two biological replicates and two experimental replicates were prepared and analyzed by iTRAQ-based HPLC-MS/MS. The samples were prepared in our laboratory using a modified protein digestion method (14). The proteins were reduced to 50 mM tris-(2-carboxyethyl) phosphine (TCEP) at 60°C for 1 h and alkylation in 200 mM Methyl methanethiosulphonate (MMTS) at room temperature for 10 min. The treated samples were diluted with 0.5 M triethylammonium bicarbonate (TEAB) to reduce the urea concentration to less than 2 M, followed by trypsin digestion at 37°C overnight with an enzyme/substrate mass ratio of 1/50. The peptides digested by trypsin were labeled by iTRAQ 8-plex reagents according to the manufacturer’s protocols (AB Sciex). Each sample was labeled separately with the iTRAQ tags: For experiment 1 (Group1), the two samples from the DK383 group were labeled with channels 114 and 115, and the two samples from the DK212 group were labeled with channels 116 and 117. Each tissue lysate was further prepared individually. A similar procedure was carried out in experiment 2 (Group2), except that the single sample was exchanged with the channels with the biological duplicate taken from groups DK383 and DK212 to eliminate the influence of tags.

### High-pH reversed-phase chromatography

iTRAQ labeled samples were redissolved with mobile phase A (20 mM HCOONH_4_, 2 M NaOH, pH 10) before HPLC on a Gemini-NX 3u C18 110A; 150×2.00 mm Phenomenex columns. The flow rate for reversed-phase column separation was 200 μl/min with mobile phase A and mobile phase B (20 mM HCOONH_4_, 80% CAN, 2 M NaOH, pH 10). A solvent gradient system was conducted: 0-15 min, 5-15% B; 15-60 min, 15-50% B; 60-80 min, 50-90% B; 80–100 min, 5% B. The UV detector was calibrated at 214/280 nm, and fractions were collected every 1 min. In total, 24 fractions were pooled for each sample and dried using a vacuum centrifuge.

### RPLC-MS analysis

A linear gradient was operated for peptide separation, which was formed from 5% ACN, 0.1% FA (mobile phase A) and 95% ACN, 0.1% FA (mobile phase B), from 5% to 40% of mobile phase B in 70 min at a flow rate of 300 nL/min. The eluted peptides were performed on a TripleTOF 5600 system (AB Sciex) in Information Dependent Mode. MS spectra were acquired across the mass range of 350–1,500 mass-to-charge ratio (m/z) in high-resolution mode (≥ 30,000) with 250 ms accumulation time per spectrum. A maximum of 20 precursors per cycle were chosen for fragmentation from each MS spectrum with 100 ms minimum accumulation time for each precursor and dynamic exclusion for 20 s. Tandem mass spectra were recorded in high sensitivity mode (resolution ≥ 15,000) with rolling collision energy on and iTRAQ reagent collision energy adjustment on, as reported previously (15). The m/z range for MS/MS scans was set from 100 to 1,500.

### Data analysis

The raw data were converted to Protein Pilot™ 4.5 (AB Sciex) equipped with the Paragon™ Algorithm (Revision Number: 4.5.0.0, 1654) for deep proteome analysis and protein quantitation. TripleTOF 5600 system performs automatic recalibration such that typical mass errors for MS and MS/MS data were below 10 ppm. The Anas platyrhynchos database (May 7, 2014, 16,588 sequences) was downloaded from NCBI, combined with common contaminants, and used for database searching. The parameters for database searching were as follows: iTRAQ 4-plex (peptide labeled) was set as sample type; MMTS was set as the cysteine alkylation; trypsin was set as the digestion enzyme; biological modifications and amino acid substitutions were set as ID focus; search effort was set as “thorough”. In Paragon™ Algorithm, iTRAQ 4-plex (peptide labeled) listed in Protein Pilot™ 4.5 were searched simultaneously with the tolerances specified as ± 0.05 Da for MS fragments and ± 0.1 Da for MS/MS fragments. A database search for the reversed database was also performed to evaluate false discovery rates (FDR) at the peptide and protein levels. Only peptide and protein identifications with FDR < 1% were retained. In addition, a strict cutoff for protein identification was controlled with an unused ProtScore ≥ 1.3, which corresponds to a confidence limit of 95%, to minimize false positive results. For iTRAQ quantitation, only peptides marked as “Auto” in Protein Pilot^TM^ 4.5 contributed to the protein ratio calculation. Bias correction was set as “Auto” and background correction as “Yes” for protein quantitation results. The coefficient of variance (CV) was calculated in biological and experimental replicates to assess the iTRAQ reliability. Proteins with an iTRAQ ratio higher than 20 or less than 0.05 were not quantified. Only proteins with at least two unique peptides and reasonable ratios across all channels were considered quantified. Protein quantitation values of Group1 and Group2 were obtained by calculating the mean of iTRAQ protein ratios of biological replicates of each group. The final protein quantitation information based on normalization to the mean of Group1 and Group2 (experimental replicates of this study) was used for further data analysis. The mass spectrometry proteomics data were deposited to the ProteomeXchange Consortium (http://proteomecentral.proteomexchange.org) with an identifier (PXD002719). Then the altered proteins were introduced into DAVID 2021 (https://david.ncifcrf.gov/home.jsp) to perform go ontology (GO) annotation and enrichment analysis. For further analysis, the duck gene identifiers of all significantly regulated proteins were converted to human protein gi numbers using BLAST of NCBI. Protein gi numbers and regulation values were imported into the Ingenuity Pathway Analysis software (IPA, www.ingenuity.com) for pathway analysis and network construction.

### Western blot

Treated cells were scraped and re-suspended in a cold RIPA buffer. Protein quantification was measured using the Pierce^®^ BCA protein assay kit (Thermo Scientific). Western blot analysis was performed as described (16) by sodium dodecyl sulfate-polyacrylamide gel electrophoresis (SDS-PAGE), using primary antibodies: anti-GAPDH (Bioworld Technology), anti-Akt (Sigma-Aldrich), anti-phosphorylated Akt (Bioworld Technology), anti-mTOR (Cell Signaling Technology), anti-phosphorylated mTOR (Cell Signaling Technology), anti-p70S6K (Santa Cruz), anti-phosphorylated p70S6K (Cell Signaling Technology), anti-phosphorylated rpS6 (Cell Signaling Technology), anti-phosphorylated 4EBP1 (Cell Signaling Technology), anti-HSP90a (Bioworld Technology), anti-desmin (Santa Cruz), anti-β-actin (Bioworld Technology), and anti-caplain-1 (Novus). Membranes were washed in TBST and incubated with DyLight488-conjugated goat anti-rabbit IgG or rabbit anti-mouse IgG (1:10000, Rockland) for 1 h. Membranes were then washed in TBST and visualized using the Odyssey Infrared Imaging system (Licor Biosciences).

### RNA and cDNA preparation

DNase-treated total RNA was isolated from lung tissue at 3 dpi using Direct-zol™ RNA MinPrep according to the instructions provided by the manufacturer (Zymo Research). Reverse transcription (RT) was conducted using the PrimeScript™ RT Reagent Kit (Takara).

### Quantitative real-time PCR (qRT-PCR)

qRT-PCR was performed using the QuantiNova™ SYBR^®^ Green PCR kit (Qiagen). Primers were designed by the software Primer3 Web (http://bioinfo.ut.ee/primer3/) based on published target sequences and previously mentioned (7, 10). Primer pairs (**Table 1**) were selected based on specificity determined by dissociation curves. The levels of PCR products were monitored using a 7500 Fast Real-Time PCR system (Applied Biosystems). PCR conditions were as follows: 95°C for 2 min, followed by 40 cycles of 95°C for 5 s, and 60°C for 32 s. Dissociation curves of the products were generated by incrementally increasing the samples’ temperature from 55°C to 100°C as the final step. For assay validation, purified products were cloned into pMD18-T (Takara) and sequenced to verify the specificity of target amplification.

**Table 1:** Primers used for qRT-PCR in this study.

Primer name	Sequence (5’-3’)	Genbank accession number
IL-6F	TTCGACGAGGAGAAATGCTT	JQ728554.1
IL-6R	CCTTATCGTCGTTGCCAGAT	
TNF-αF	ATGAACCCTCCTCCGTACAC	EU375296.1
TNF-αR	TCTGAACTGGGCGGTCATAA	
IL-10F	GGGGAGAGGAAACTGAGAGATG	JN786941.1
IL-10R	TCACTGGAGGGTAAAATGCAGA	

### Statistics analysis

Expression fold change of target genes in the infected group versus those in the control group was determined by the 2^−ΔΔCt^ method using the duck housekeeping gene glyceraldehyde-3-phosphate-dehydrogenase (GAPDH) as the endogenous reference gene to normalize the level of target gene expression (10). Standard deviations were calculated using the fold change values of three replicates for each gene. Comparisons on virus titer in lung tissues and qRT-PCR data were conducted for statistical analyses by GraphPad Prism 5 software (GraphPad Software, Inc.).

## Results

**Figure 1:**
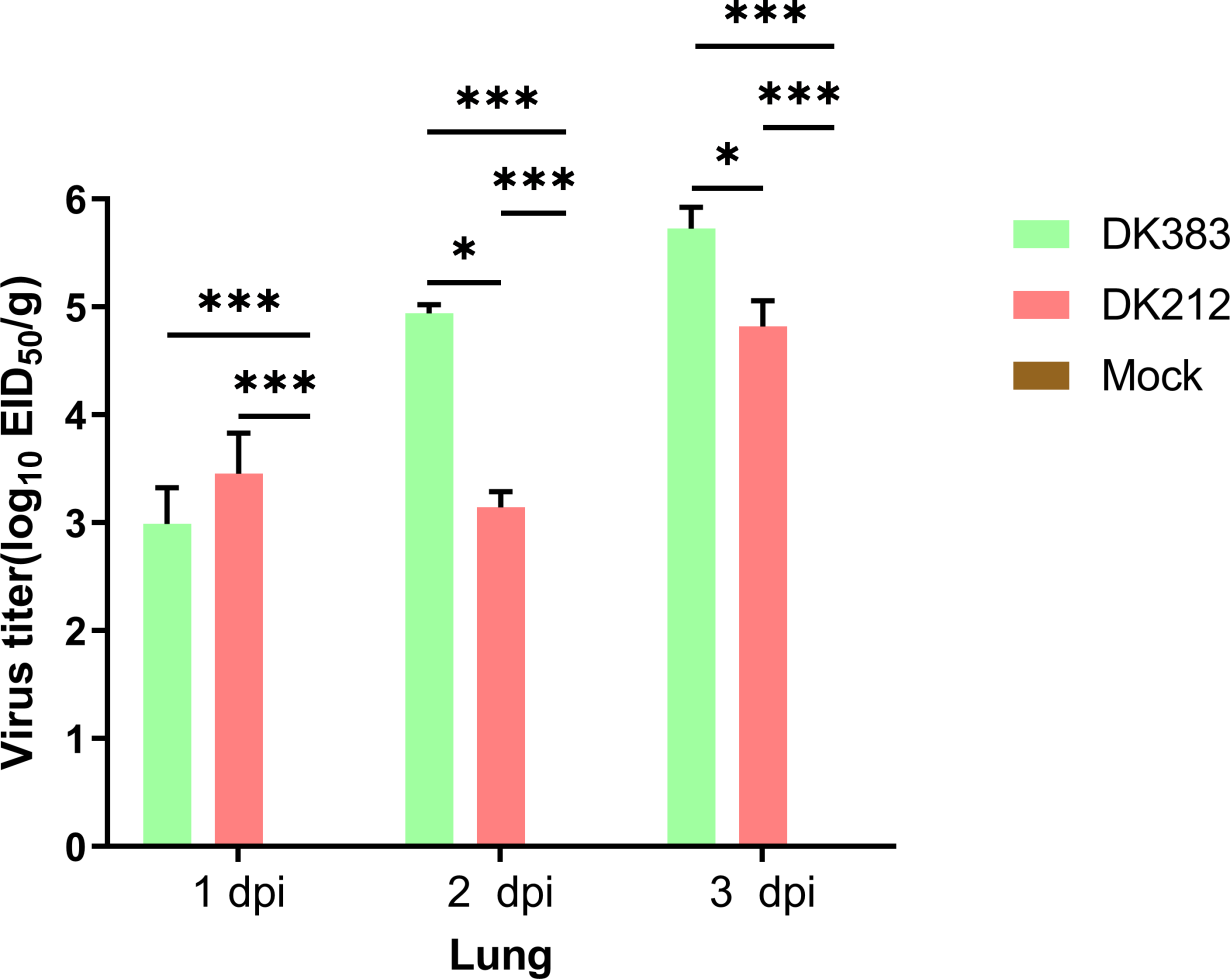
Virus titers in the lung of Muscovy ducks infected with DK383 and DK212 at 1, 2, and 3 dpi. Virus titers were calculated as means of standard deviation in log10 EID_50_/g of tissue. * and *** indicated significant differences with *P* ≤ 0.05 and *P* ≤ 0.001.

In this study, the ducks were infected separately by two H5N1 HPAI strains, DK383 or DK212, and initially used iTRAQ 8-plex reagent to investigate the host proteome response to the H5N1 HPAI virus. To compare disease progression, samples from 1, 2, and 3 dpi were selected to evaluate virus load. DK383 replicated to a much greater extent within the lungs than DK212 and reached the peak, particularly at 3 dpi. This result was consistent with our previous results (**Figure 1**). DK383 could cause a more productive infection and replicate more efficiently than DK212 (10, 12). Ducks in the DK383 group displayed more severe symptoms than those in the DK212 groups within 3 dpi. Signs included hemorrhages of the beak, torticollis, anorexia, depression, neurological symptoms on day 3 p.i., and death on 4 dpi. However, no ducks exposed to DK212 died or showed clinical signs of infection. Altogether, lung tissues of DK383 and DK212 at 3 dpi were designated for later proteomics analysis.

### Reproducibility assessment of protein identification among replicates and determination of the cutoff value

Two independent iTRAQ experiments were conducted as experimental replicates on day 3 p.i. in different virulence of H5N1 influenza virus infection. Two animals of each group were analyzed in separate iTRAQ channels as biological replicates. Peptides and proteins were retained according to the values of FDR and unused ProtScore. We then assessed biological and experimental replicates by calculating the coefficient of variance (CV) (17) when the recommended cutoff point of biological replicate is at ± 50% variation, the coverages of Group1 and Group2 in protein quantification were 86% and 92%, respectively (**Supplementary Figure 1**). While the cutoff point at ± 50% variation for experimental replicate between Group1 and Group2, an 86% coverage interval in protein quantification was recorded (**Supplementary Figure 2**), which indicated greater proteome coverage and precise quantification (18).

**Figure 2:**
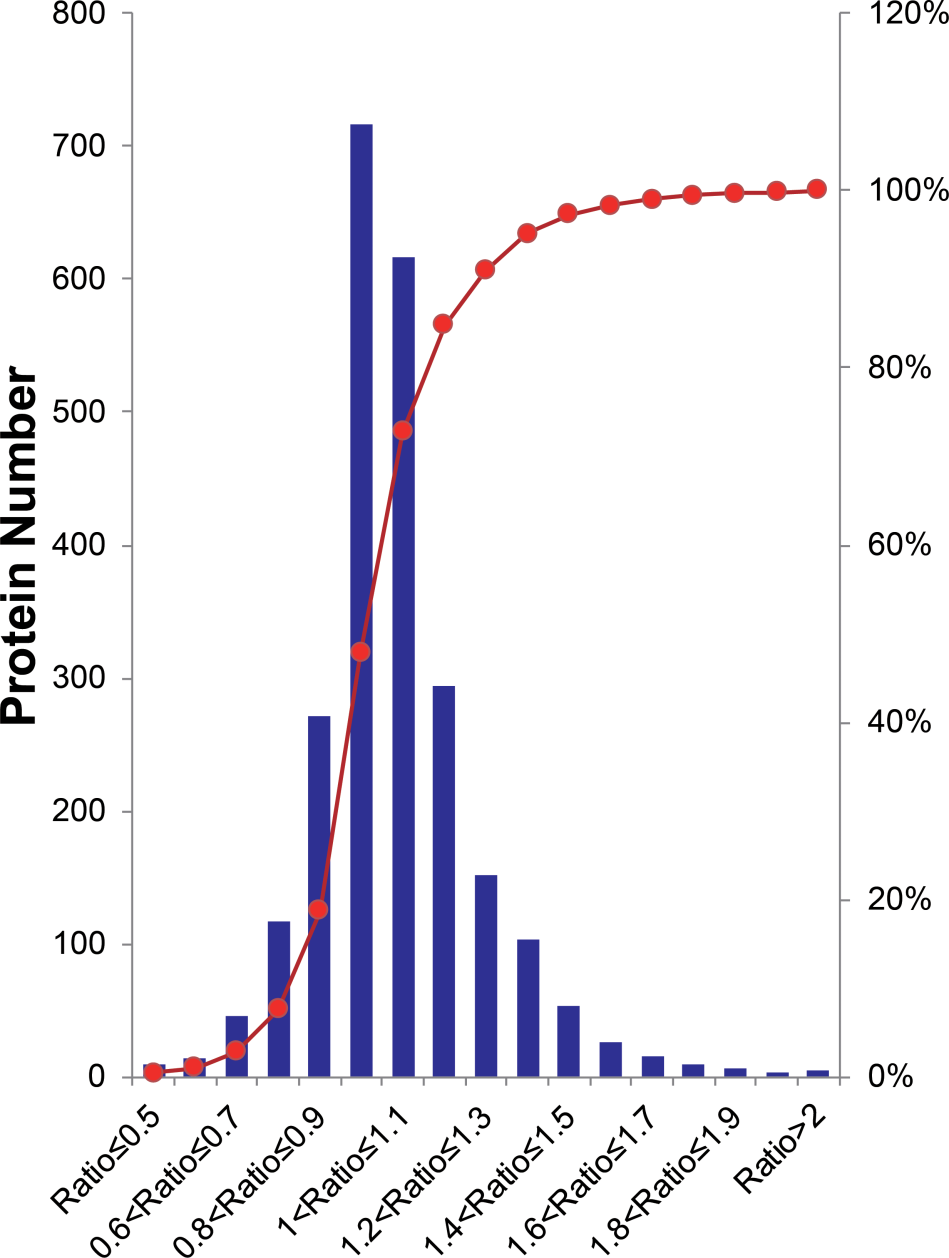
Histogram distribution of expression ratios of quantified proteins. The Red line indicates the cumulative percentage of proteins.

Overall, peptides from biological and experimental replicates were pooled and characterized using a cutoff CV of 0.5. iTRAQ ratios of protein expression higher than 20 or lower than 0.05 were not considered in the further analysis. Altogether, 16,680 peptides were acquired in the two independent experiments, of which 74.6% of Group1 and 64.8% of Group2 were detected. Correspondingly, 3,000 proteins were identified, in which 90.5% of Group1 and 82.3% of Group2 were detected. From this total, 2,459 proteins were quantified across both experimental replicates, in which 89.6% of the Group1 and 94.4% of Group2 were detected (**Supplementary Figure 3**), for a final 85.1% reproducibility, implying that there were high correlation rates between both biological replicates and experimental replicates. The quantified proteins in two replicates fit a Gaussian distribution based on the ratios (**Figure 2**). The ratio distribution was narrow, with a mean of 1.034 and a standard deviation (SD) of 0.1986. According to traditional statistics, 0.6107 and 1.389 were considered significant differences (*P* ≤ 0.05), which were located at the lower and upper bound, respectively. To reduce the omission of some valuable information, we slightly expanded the range of analysis. Eventually, proteins with expression ratios ≥ 1.3 or ≤ 0.7 were recognized as significantly up- and down-regulated, respectively. As a result, 297 proteins were differentially expressed, including 222 up-regulated and 70 down-regulated proteins.

### Bioinformatics analysis of overexpressed proteins

**Figure 3:**
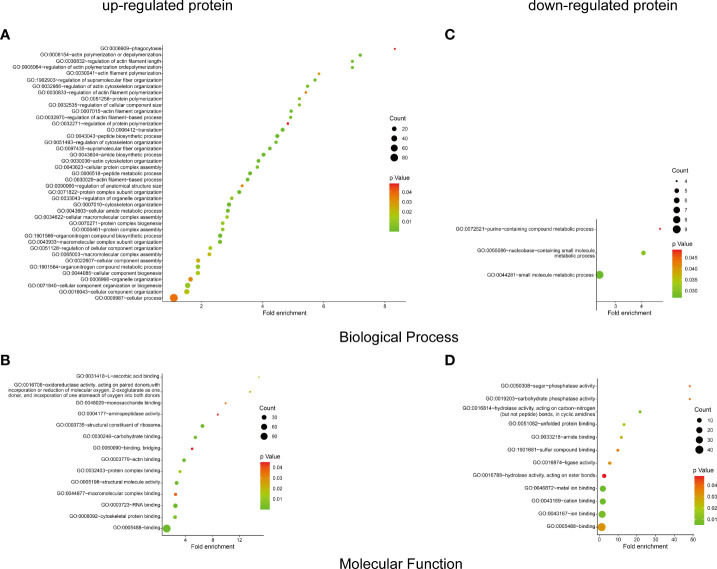
GO enrichment analysis of the differential proteins. The biological process and molecular function terms enrichment results of up-proteins **(A, B)** and down-regulated proteins **(C, D)** were conducted by DAVID. More information is available in **Supplementary Table 1**. The figure was created with ImageGP (http://www.ehbio.com/ImageGP/).

**Figure 4:**
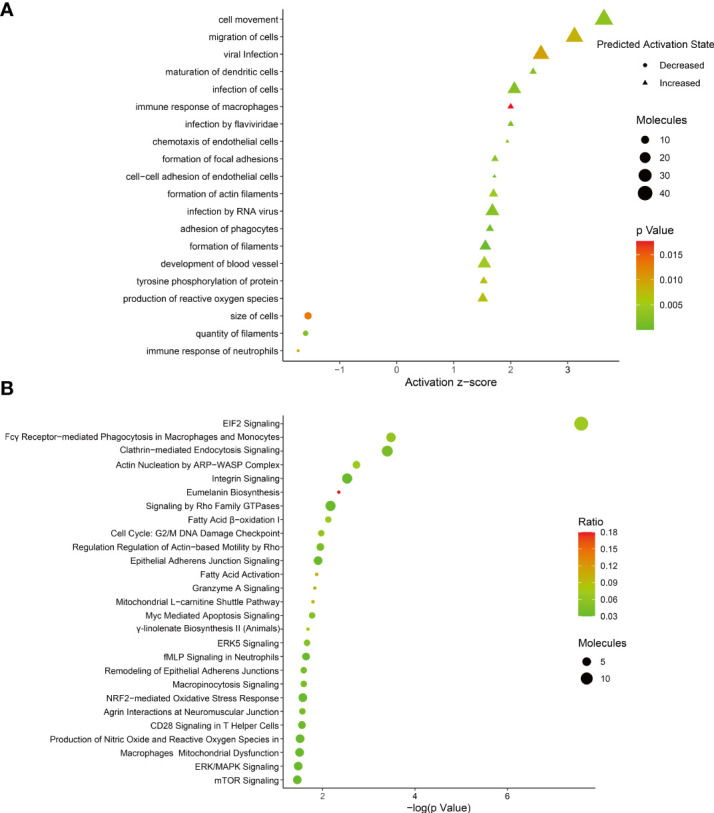
**(A)** bio-function analysis results of proteins with differential expression. The circle indicates increased effects on the function predicated by IPA, and the triangle indicates that the biological process or disease is trending towards a decrease; **(B)** enrichment of canonical pathways within each category was determined using IPA software. More information is available in **Supplementary Table 2**. The figure was created with ImageGP.

To identify the host protein function manipulated by two H5N1 HPAI strains in this study, GO term enrichment analysis of the differentially expressed genes was carried out. In biological process terms, the up-regulated proteins may involve in phagocytosis, actin polymerization or depolymerization, translation, and peptide biosynthetic process, while the down-regulated proteins only may participate in purine-containing compound metabolic process, purine-containing compound metabolic process, and small molecule metabolic process. In molecular function terms, the L-ascorbic acid binding, monosaccharide binding, and structural constituent of ribosome were enriched in the up-regulated proteins, whereas sugar-phosphatase activity, carbohydrate phosphatase activity, and unfolded protein binding were enrichment in the down-regulated proteins (*P* ≤ 0.05, **Figure 3** and **Supplementary Table 1**). Since the duck genome database has inadequate annotation by contrast with the human genome, many proteins are uncharacterized or unknown. Each identifier of the altered proteins in **Supplementary Table 1** was converted to its corresponding human protein gi number. Protein gi numbers and expression ratios were imported into the IPA tool, and a global molecular network and pathway were then developed from information previously reported based on the underlying biological evidence, such as protein interactions, regulation of expression, etc. We discovered that the previously characterized proteins were assigned to 26 diseases or functions annotation. This bio function classification was identified at statistically significant levels (*P* ≤ 0.05) displayed in **Figure 4A**, with additional data shown in **Supplementary Table 2**. The multiple protein clusters were predicated on being activated or up-regulated in the DK212 group compared with the DK383 group, including cell movement, maturation of dendritic cells, immune response of macrophages, chemotaxis of endothelial cells, and adhesion of phagocytes. By contrast, proteins in these clusters were predicated on being up-regulated in the DK383, including the immune response of neutrophils, the number of filaments, and the size of cells. The most relevant canonical pathways following the influenza virus infection included eIF2 signaling, Fcγ receptor-mediated phagocytosis in macrophages and monocytes, CD28 signaling in T helper cells, production of nitric oxide, and reactive oxygen species in macrophages, mitochondrial dysfunction, and mTOR signaling. (*P* ≤ 0.05, **Figure 4B** and **Supplementary Table 2**). It is suggested that the H5N1 virus may involve manipulation and interference with immune regulatory mechanisms.

**Figure 5:**
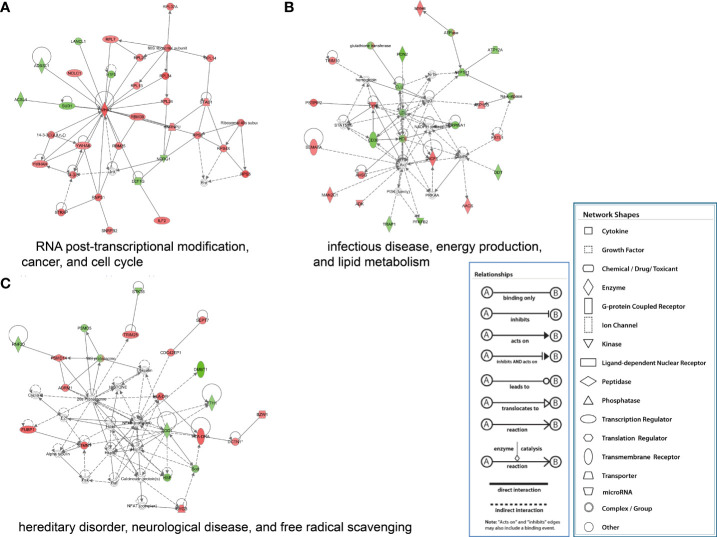
IPA of proteins that were significantly altered to construct specific functional networks. Red, up-regulated proteins; green, down-regulated proteins; white, proteins known to be in the Ingenuity Pathways Knowledge Base but were not identified in this study. The color depth indicates the magnitude of the change in protein expression level. The shapes indicate the molecular class (i.e., protein family). Lines connecting the molecules imply molecular relationships. Dashed lines indicate indirect interactions, and solid lines indicate direct interactions. The arrow styles mean specific molecular relationships and the directionality of the interaction. **(A)** RNA posttranscriptional modification, cancer, and cell cycle; **(B)** infectious disease, energy production, and lipid metabolism; **(C)** hereditary disorder, neurological disease, and free radical scavenging.

The proteins significantly regulated were subject to regulatory network analysis, which may further the investigation of possible links between influenza virus infection and diseases such as acute lung injury. Network analysis suggested these proteins were mapped to 10 specific functional networks (**Figure 5** and **Supplementary Table 2**). The three networks of interest correspond to (1) RNA posttranscriptional modification, cancer, and cell cycle; (2) infectious disease, energy production, and lipid metabolism; (3) hereditary disorder, neurological disease, and free radical scavenging.

### Validation of identified candidates by western blot

**Figure 6:**
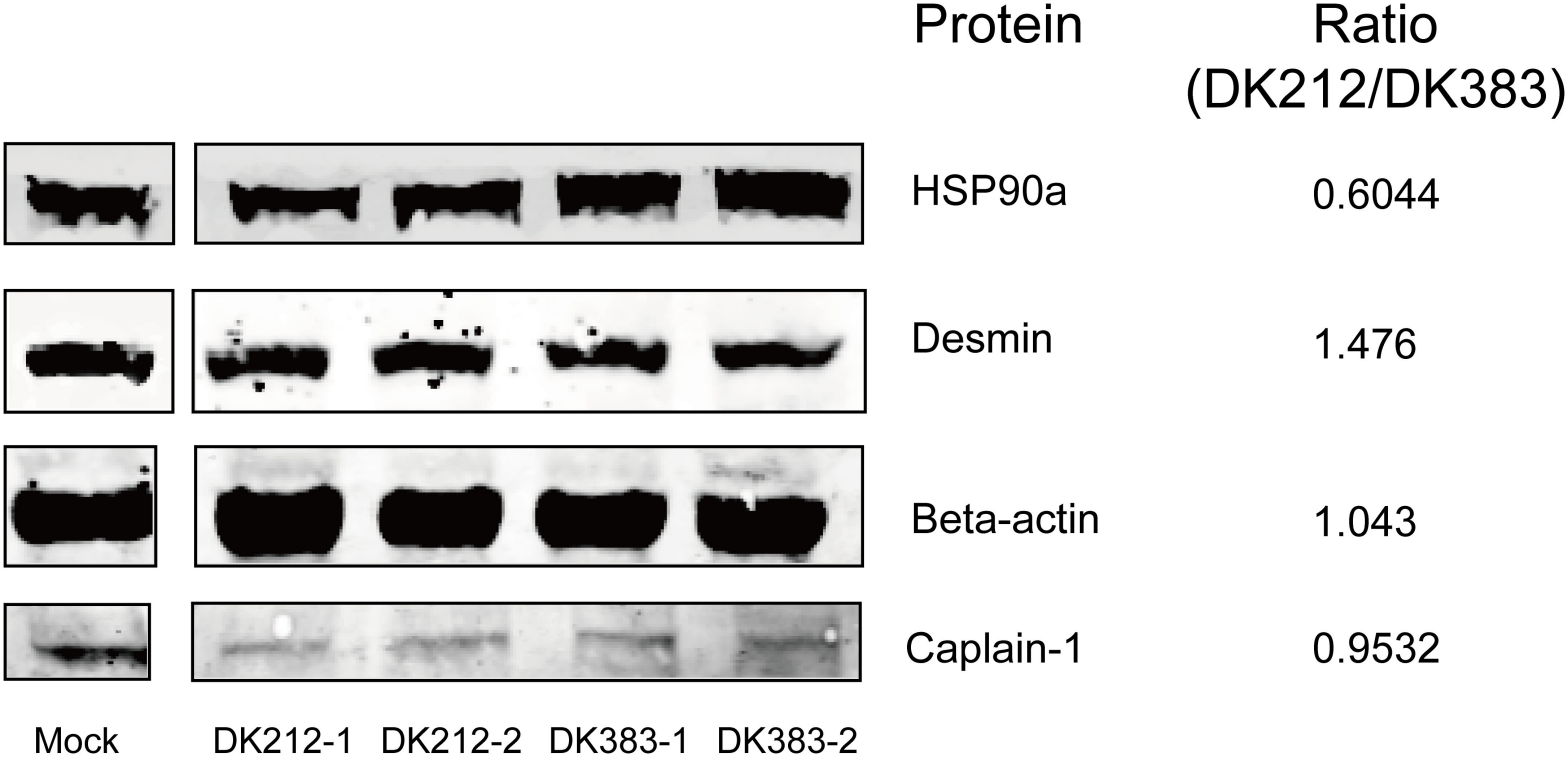
Western blot analyses of the representative proteins. Ratios of each protein between DK212 and DK383 were determined by iTRAQ. Compared with the DK383 group, decreased expression was observed for HSP90a, increased expression was found for desmin, and the expression of β-actin and caplain-1 were not altered in the DK212 group.

To confirm the protein quantification, immunoblotting analyses were carried out on a series of protein candidates extracted from lung tissues of DK383 and DK212 ducks. Although there are insufficient antibodies to be appropriately used for the quantified proteins, the results in **Figure 6** demonstrated the ratios of the four representative proteins (HSP90a, desmin, β-actin, and caplain-1) were consistent with those data obtained from iTRAQ-identified candidates.

### Akt/mTOR/p70S6K pathway is involved in the duck proteome response to influenza virus infection

**Figure 7:**
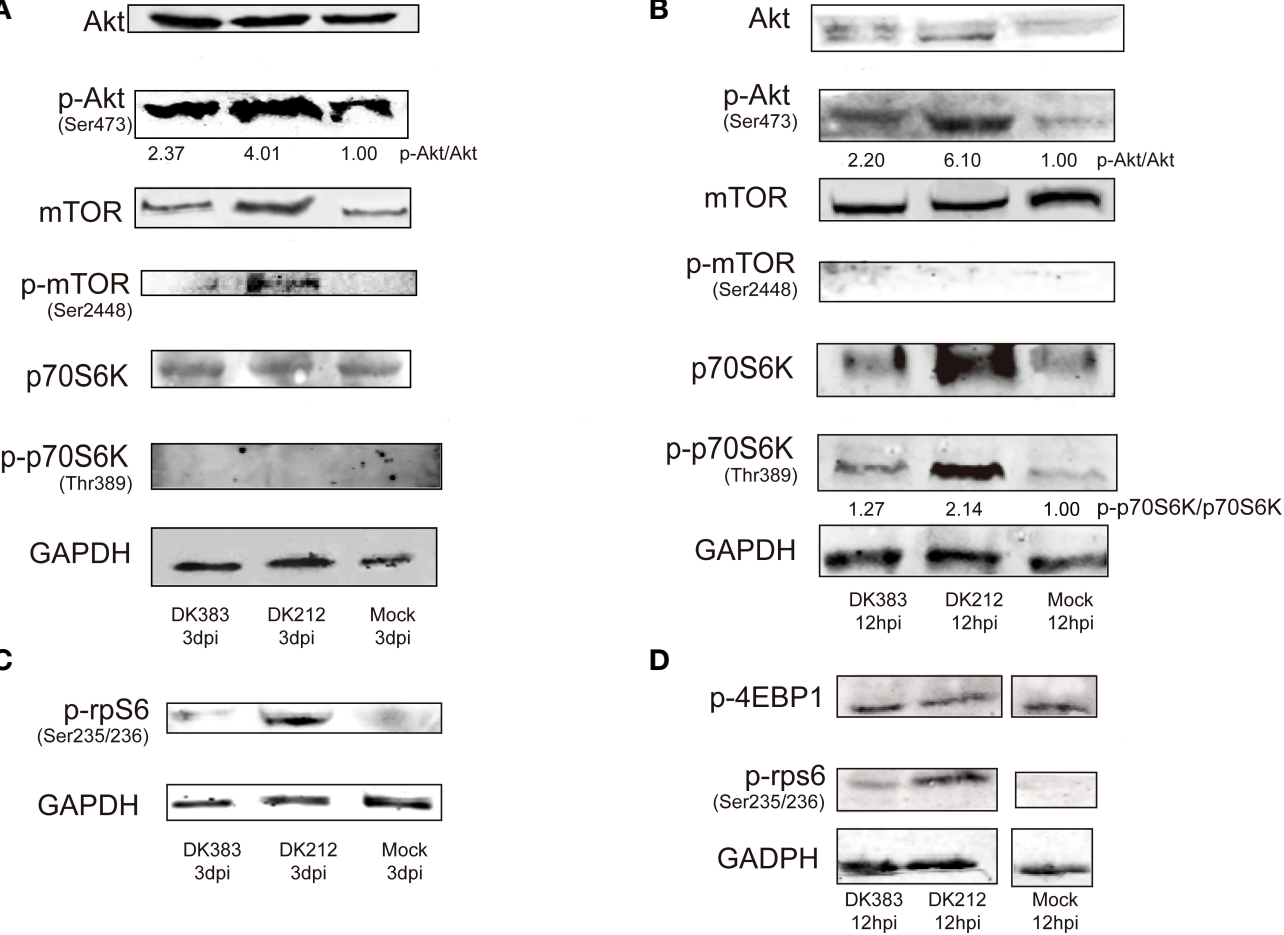
Immunoblot analysis of mTOR pathway activation in duck lung tissues at 3 dpi DEF lysates at 12 hpi infected with DK383 or DK212. Total and phosphorylation levels of Akt, mTOR, and p70S6K are shown in lung tissues **(A)** and DEF **(B)**; **(C)** shows phosphorylation of rpS6 in lung tissues; **(D)** shows phosphorylation of 4EBP1 and rpS6 in infected DEF. Densitometry of signal intensity of the phosphorylation protein normalized to the corresponding total protein was labeled under each band. The mock-infected group was used as the negative control.

As mentioned above, mTOR signaling was highlighted in the canonical pathway analysis. Several proteins in this pathway formed a group whose abundance differed between DK212 and DK383 infected ducks. For example, eIF3M, RPS8, RPS5, and RPS4X are downstream elements of mTOR signaling (**Supplementary Table 2**). The proteins were up-regulated in the DK212 group, indicating that mTOR signaling may be promoted more obviously than in the DK383 group. As reported previously, moderate mTOR phosphorylation induces a protective effect in influenza virus-infected cells (13). Because the sample pooling strategy could reduce the biological variation, increasing the power to detect changes (19). For each condition, pooled samples consisting of three biological replicates were collected. To measure whether members of mTOR signaling were activated in H5N1 virus-infected lung tissues, lysates from these pooled samples were evaluated by western blot. As illustrated in **Figure 7**, robust phosphorylation in Akt (Ser473) and mTOR (Ser2448) were observed after the duck lung tissues were treated with DK383 and DK212 at 3 dpi compared with the control, which demonstrated the activation of the Akt/mTOR pathway. p70S6K is considered an important component of mTOR signaling, and our results demonstrated that phosphorylation in p70S6K (Thr389) was not detected.

However, rpS6, downstream of p70S6K, showed enhanced phosphorylation. DK212 infection dramatically induced phosphorylation of rpS6 compared with DK383. This phenomenon indicated facilitating the initiation of mRNA translation and further confirmed the activation of the mTOR pathway. These findings were further validated by comparing them to these proteins of mTOR signaling in influenza-infected DEF. As expected, we observed that DK212 and DK383 both markedly increased the levels of p-Akt (Ser473), p-p70S6K (Thr389), and p-rpS6 (Ser235/236). When the cells were treated with DK212, these phosphorylated proteins significantly increased too much higher than those observed in cells treated with DK383 (**Figure 7**). Meanwhile, there was no significant difference between each group in phosphorylated 4EBP1, the other substrate of mTOR. Therefore, amplified phosphorylation of proteins in the Akt/mTOR/p70S6K pathway revealed a host response that negatively regulated this signaling cascade after infection by a virulent H5N1 virus.

### Akt/mTOR/p70S6K pathway controls cytokine bias

**Figure 8:**
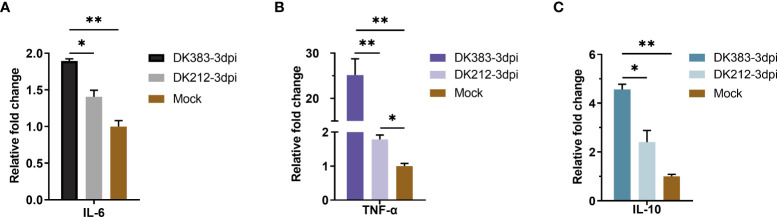
qPCR expression analysis of proinflammatory cytokine IL-6 **(A)** and TNF-α **(B)** and anti-inflammatory cytokine IL-10 **(C)** in the lungs of infected duck with the two HPAIVs at 3 dpi. Gene expression was normalized to the GAPDH gene expression level and is presented as the fold change relative to the level for the control group. Error bars indicate standard deviations. (*) *P* ≤ 0.05 and (**) *P* ≤ 0.01 compared with the result for DK212, respectively.

As we all know, mTOR complexes regulate metabolic processes of cells, including protein synthesis (20). Innate or adaptive immune signals could trigger the Akt-mTOR-p70S6K signaling cascade in innate immune cells. mTOR activation can inhibit the proinflammatory molecules, such as IL-6 and tumor necrosis factor-alpha (TNF-α), and also boost the production of anti-inflammatory cytokines such as interleukin-10 (IL-10) (21). Due to a limited number of appropriate reagents to measure these cytokines of ducks in the serum, mRNA levels of the core proteins were used to evaluate by qRT-PCR. A significant increase in mRNA levels of IL-6 was observed at 3 dpi, and the IL-6 expression in DK383 infected group was higher than that of the DK212 infected group, which was previously described (10). A similar pattern was found in TNF-α mRNA levels after infection by the virulent H5N1 virus DK383. However, the level of IL-10 mRNA was remarkably lower in DK212 infected lung tissue (**Figure 8**). As reported in the earlier study (22), the differences in cytokine production did not correlate with gene expression. Hence, the cytokine biasing reported in response to virulent H5N1 virus may involve posttranscriptional gene expression regulation.

## Discussion

Prior work has focused on the host response to influenza virus infection in RNA expression profiles within the duck model. However, several proteomic analyses have been implemented in the influenza virus-host interactomes. This study provided a comprehensive analysis describing the host proteomes of ducks during infection with the H5N1 virus. An unbiased strategy-iTRAQ, combined with HPLC-MS/MS, enhanced the proteome coverage and protein specificity. The duck genome database annotation development also facilitated the broadened identification of peptides and proteins (11). H5N1 viruses have employed various strategies to regulate innate and adaptive immune responses to establish productive infections in an individual. While proteins involved in the immune response of neutrophils and the size of cells were elevated significantly in the lung by the virulent virus DK383, the avirulent virus DK212 induced a distinct response characterized by an increase in proteins that participated in cell movement, maturation of dendritic cells, adhesion of phagocytes, and immune response of macrophages.

There is increasing evidence for a critical role in the “maturation of dendritic cells” at the onset of antiviral immunity. Contrasting with a seasonal virus, the pandemic H1N109 influenza virus failed to induce substantial dendritic cell maturation (23). DK212 infection substantially repressed CD36, THBS1, and SERPINA1 expression, which might prevent the maturation of immature dendritic cells. ITGB2, LYN, and STAT1 were up-regulated by comparison with DK383, suggesting that the virulent virus might impede this biological process. Compared with low pathogenic H1N1 (PR8) in infected human monocyte-derived macrophages, the H5N1 HPAI virus revealed an insufficient innate immune response. This strategy contributes to virus spreading and progression to the systemic stage of disease (24). The bio-function “adhesion of phagocytes”, “cell movement”, and “immune response of macrophages” were significantly regulated in DK212, which indicated the avirulent virus might have a positive effect on the immune response to eliminating pathogen. Moreover, the neutrophils ameliorate lung injury and facilitate the development of severe disease during influenza infection (25). Thus, the categories “immune response of neutrophils” and “size of cells” were dramatically elevated, which may aggravate the tissue injury.

To mine our data, we applied IPA software to determine which signaling pathways were enriched in each group. We discovered that multiple members in the pathways of eIF2 signaling, Fcγ receptor-mediated phagocytosis in macrophages and monocytes, and CD28 signaling in T helper cells were up-regulated in DK212. In eukaryotic cells, induction of the eIF2 signaling pathway occurs following infection with pathogens. Bacterial virulence factor YopJ reduced eIF2 signaling to repress proinflammatory cytokine expression and be susceptible to bacterial invasion. Viral infection also activates eIF2-mediated translation control. This effect causes a decrease in general protein synthesis and reduces viral replication while enhancing the translation of specific stress-related mRNA transcripts, such as ATF3 and CHOP. The highly conserved eIF2 signaling pathway is vital for antiviral responses (26). Meanwhile, Fc receptor-mediated phagocytosis plays a pivotal role in the clearance of influenza virus infections (27). The costimulatory pathway CD28 signaling is essential for activating helper T cells and protective immunity to influenza infection (28). Taken together, it appears that down-regulation of these pathways may promote the evasion of the host immune responses for productive infection in DK383.

The mTOR signaling pathway was up-regulated in DK212 compared with DK383. Akt/mTOR/p70S6K pathway plays a vital role in regulating immune function and protein translation in response to environmental stress, such as infection. Our data indicated that infection of lung tissue and DEF with either a highly virulent or avirulent H5N1 virus favored ubiquitinylation of Akt, mTOR, p70S6K, and rpS6. The avirulent virus-induced mTOR pathway more noticeably than the virulent virus indicates that this pathway was differentially regulated in response to influenza infection. In the previous research, alterations in phosphorylation of the PI3K/Akt/mTOR signaling pathway suggests that influenza viruses highjack the host response responsible for aiding viral replication and pathogenesis (29). Suppression of mTOR function inhibited translation and boosted proinflammatory cytokine secretion (22). Influenza A virus NS1, a known multifunction protein, has been reported to activate Akt phosphorylation (30). This phenomenon indicates NS1 may serve as an inducer of mTOR. However, NS1 has a negligible influence on the accumulation of mTOR in the H1N1-infected cells (31). In the duck model, it is unknown whether the regulator of NS1 in cells infected with the H5N1 virus is analogous to the effect of H1N1. Therefore, further work should be directed to understand the precise regulatory mechanism better.

To gain a more detailed understanding of changes in host response proteome during H5N1 virus infection, we focused on an early infection event about interferon (IFN) response by searching the known interferon-stimulated genes to determine which might be associated with influenza virus infection. Influenza virus NS1 protein can impair antiviral IFN response by antagonizing RIG-I’s ubiquitination (32). Our results demonstrated that many tripartite motif-containing proteins (TRIM) were up-regulated in the DK212, which could mediate ubiquitination and enhance innate immune signaling, including TRIM10, 25, and 47. Most importantly, TRIM25 can augment the production of biologically functional, antiviral cytokines and reduce virus replication (33). Meanwhile, the expression of the final effector STAT1 in the IFN response pathway was markedly increased at 3 dpi. It may be reasonable to assume that the DK212 group could have induced more robust antiviral immunity than the DK383 group.

In contrast to Lys172 residue of human RIG-I critical for efficient TRIM25-mediated ubiquitination, duck RIG-I activated by TRIM25 is independent of anchored ubiquitin (34, 35). However, little is known about whether TRIM25 was regulated differentially by NS1 proteins of various virulent strains. This finding is promising and should be explored in the next stage. Not all genomic and proteomic data were in good agreement (36). Discrepancies that are always difficult to explain might provide objective answers concerning influenza virus pathogenesis. Future advances in biological system analyses integrating these approaches will potentially refine our understanding of the influenza virus infection.

In conclusion, in the present work, an iTRAQ-based quantitative proteomics strategy was developed to dissect Muscovy duck lung tissue infected by H5N1 HPAI viruses to quantify changes in host protein expression. Bioinformatics analysis of these proteomics data suggested multiple signaling pathways may be involved in the host response to H5N1 HPAI viruses, which provide insights into the mechanisms of infection. To validate our results, Akt/mTOR/p70S6K pathway was confirmed to be activated during the infection of H5N1 HPAI viruses. In summary, our data highlights the interaction between the H5N1 HPAI virus and the host and may help to elucidate the potential mechanism of pathogenicity and inflammation.

## Data availability statement

The data presented in the study are deposited in the ProteomeXchange Consortium repository (http://proteomecentral.proteomexchange.org), accession number PXD002719.

## Ethics statement

The animal study was reviewed and approved by ABSL-3 Committee of South China Agricultural University.

## Author contributions

HF and ML conceived the study. YY, QL, ZZ, PM, JuZ, JL, and JiZ performed the experiments. YY and HF analyzed the data. YY wrote the draft. HF and ML revised the manuscript. All authors contributed to the article and approved the submitted version.

## Funding

This work was supported by the Natural Science Foundation of Guangdong Province, China (2014A030313462), Science & technology nova Program of Pearl River of Guangzhou (2012J2200086), Programs for Science and Technology Development on H7N9 Avian Influenza of Guangdong Province (20140224), the China Agricultural Research System (CARS-42-G09), Joint Research Projects on H7N9 Influenza ((2014) no.1046), and the Modern Agriculture Talents Support Program ((2012) no.160).

## Acknowledgments

We thank Hejia Ye for the technical assistance in the animal experiment.

## Conflict of interest

The authors declare that the research was conducted without any commercial or financial relationships construed as a potential conflict of interest.

## Publisher’s note

All claims expressed in this article are solely those of the authors and do not necessarily represent those of their affiliated organizations, or those of the publisher, the editors and the reviewers. Any product that may be evaluated in this article, or claim that may be made by its manufacturer, is not guaranteed or endorsed by the publisher.
