# Supplementary material for: Differential proteome response to H5N1 highly pathogenic avian influenza (HPAI) viruses infection in duck
Source: Front Immunol. 2022 Aug 19;13:965454. doi: 10.3389/fimmu.2022.965454 (PMC9438030; doi:10.3389/fimmu.2022.965454)
Supplement: Supplementary file 1 [file DataSheet_1.docx]

***Supplementary Material***

## Supplementary Tables

**Supplementary Table 1**. Table of all proteins quantification summary by mass spectrometry for Group1 and Group2 and GO clustering of the differential proteins.

**Supplementary Table 2**. Bioinformatics analysis of differential proteins by Ingenuity Pathway Analysis.

**Supplementary Table 3**. iTRAQ-based peptide Summary report for Group1.

**Supplementary Table 4**. iTRAQ-based peptide Summary report for Group2.

**Supplementary Table 5**. iTRAQ-based protein Summary report for Group1 and Group2.

## Note: The size of Table 3 and 4 are large than 30 MB. Therefore, the files cannot be uploaded through submission. Table 3 and 4 were deposited to a cloud drive (http://pan.baidu.com/s/1nvi4sRn, password: y23x), which are freely available for academic research.

## Supplementary Figures


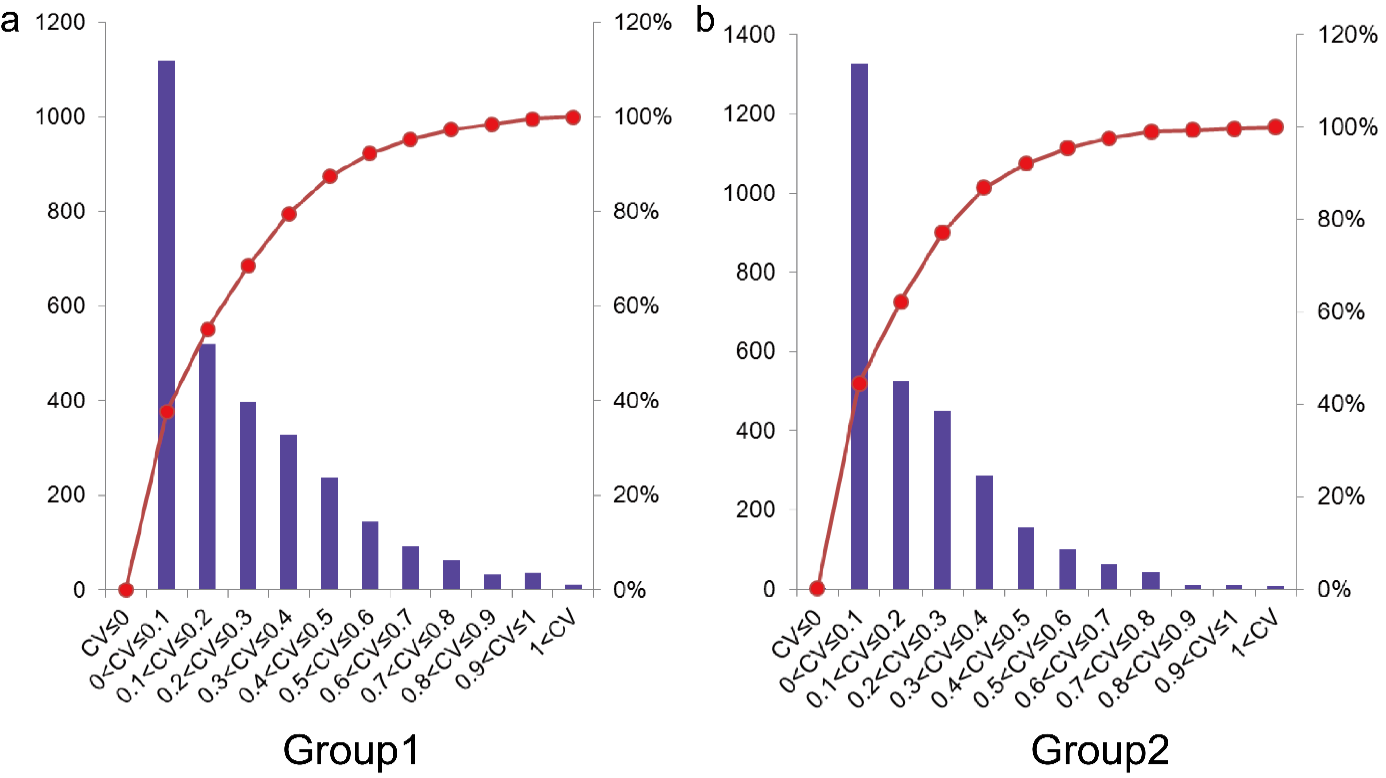


**Supplementary Figure 1**. The percentage coverage at a different variation level for biological replicates in Group1 (a) and Group2 (b). Red line indicates cumulative percentage of proteins.


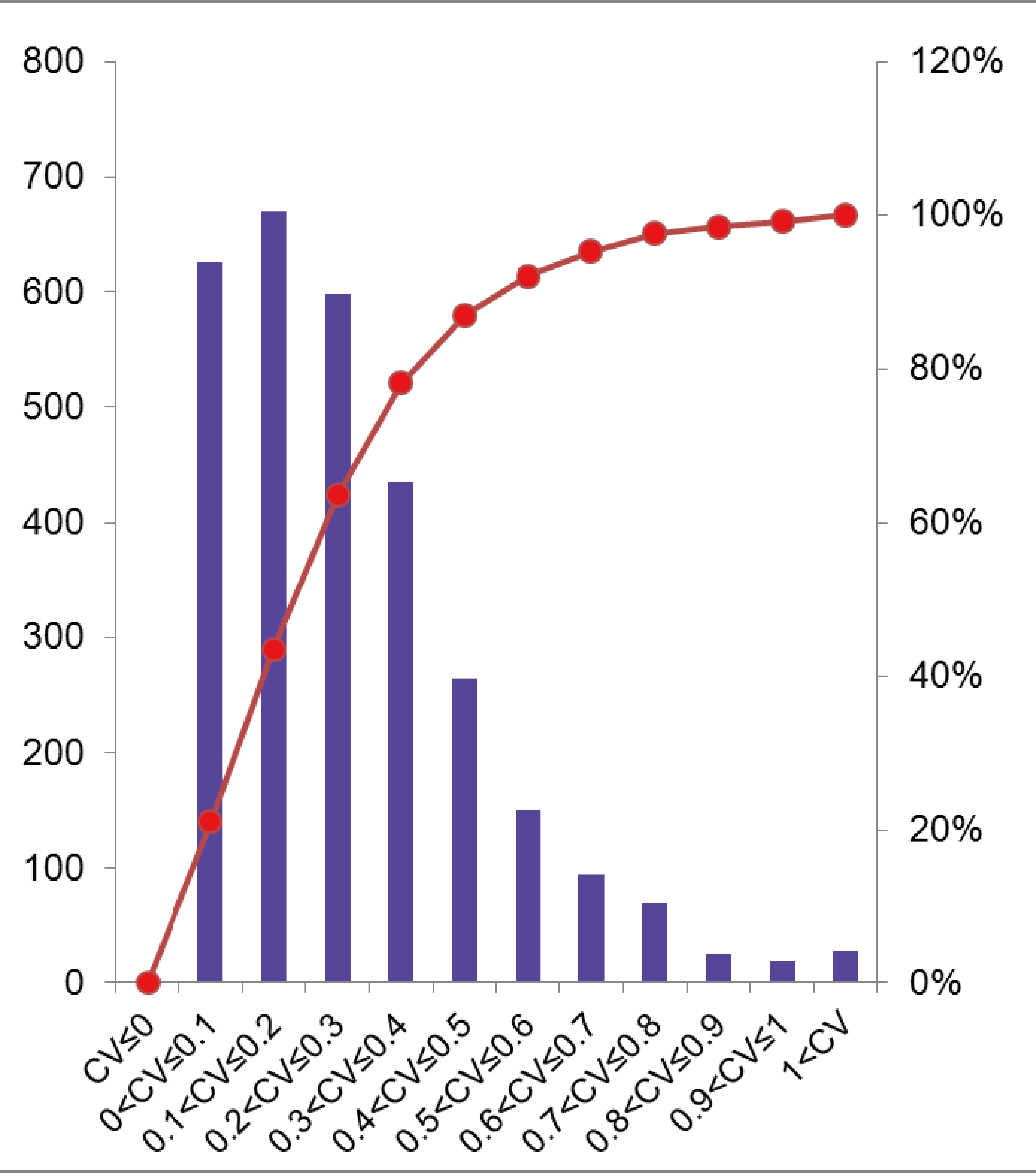


**Supplementary Figure 2.** The percentage coverage at different variation levels for experimental replicates. Red line indicates cumulative percentage of proteins.


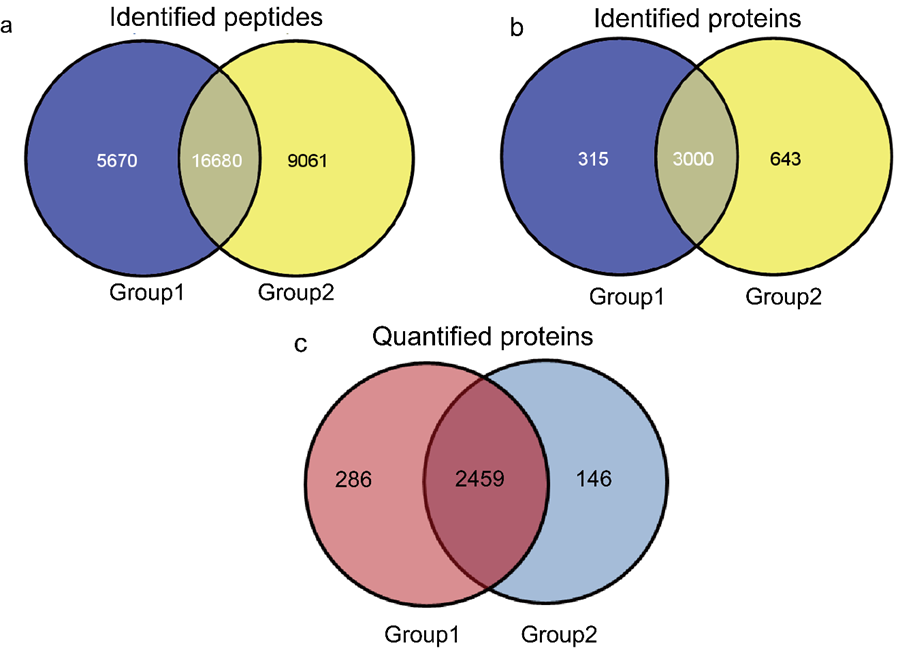


**Supplementary Figure 3**. (a) Venn diagram quantifying the intersection of peptides from two experimental replicates. As shown, 16,680 peptides were identified in the two independent experiments, 74.6% (16,680/22,350) of Group1 peptides and 64.8% (16,680/25,741) of Group2 peptides were detected. (b) Venn diagram comparing numbers of protein identifications of two experimental replicates. 3,000 proteins were commonly found, 90.5% (3,000/3,315) of the Group1 and 82.3% (3,000/3,643) of the Group2 were detected, respectively. (c) Venn diagram summarizing numbers of quantified protein identification overlaps between two experimental replicates. 2,459 proteins were quantified in two runs, 89.6% (2,459/2,745) of the Group1 and 94.4% (2,459/2,605) of the Group2 were detected.
